# Simultaneous Analysis of Anthocyanin and Non-Anthocyanin Flavonoid in Various Tissues of Different Lotus (*Nelumbo*) Cultivars by HPLC-DAD-ESI-MS^n^

**DOI:** 10.1371/journal.pone.0062291

**Published:** 2013-04-30

**Authors:** Sha Chen, Yue Xiang, Jiao Deng, Yanling Liu, Shaohua Li

**Affiliations:** 1 Key Laboratory of Plant Germplasm Enhancement and Speciality Agriculture, Wuhan Botanical Garden, Chinese Academy of Sciences, Wuhan, P. R. China; 2 Beijing Key Laboratory of Grape Science and Enology, and Laboratory of Plant Resource, Institute of Botany, Chinese Academy of Sciences, Beijing, P. R. China; 3 University of Chinese Academy of Sciences, Beijing, P. R. China; Enzo Life Sciences, Inc., United States of America

## Abstract

A validated HPLC-DAD-ESI-MS^n^ method for the analysis of non-anthocyanin flavonoids was applied to nine different tissues of twelve lotus genotypes of *Nelumbo nucifera* and *N. lutea*, together with an optimized anthocyanin extraction and separation protocol for lotus petals. A total of five anthocyanins and twenty non-anthocyanin flavonoids was identified and quantified. Flavonoid contents and compositions varied with cultivar and tissue and were used as a basis to divide tissues into three groups characterized by kaempferol and quercetin derivatives. Influences on flower petal coloration were investigated by principal components analyses. High contents of kaempferol glycosides were detected in the petals of *N. nucifera* while high quercetin glycoside concentrations occurred in *N. lutea*. Based on these results, biosynthetic pathways leading to specific compounds in lotus tissues are deduced through metabolomic analysis of different genotypes and tissues and correlations among flavonoid compounds.

## Introduction

Lotus (*Nelumo*) is in use in China not only as an ornamental plant but also as an important aquatic vegetable [1]. Various lotus tissues rich in flavonoids are employed in traditional Chinese medicine as well as serving as foodstuffs [2]–[7]. All lotuses worldwide belong to two species, *N*. *nucifera* and *N*. *lutea*. The former, also named Chinese lotus and rich in germplasm variants, exists as at least 600 cultivars [8] consisting of three types (seed, flower, or rhizome-producing) based on their use and morphological differences [9]. Lotus flowers come in a variety of bright colors, including red, pink, white with red borders, plain white (*N*. *nucifera*), and yellow (*N*. *lutea*) [8]. The study of the pigments of lotuses with different genetic backgrounds may provide information to clarify the formation of flower colors.

Flavonoids are natural antioxidant constituents of the human diet and have been receiving special attention due to their health-maintaining properties. Their antioxidative effects make flavonoids useful in a number of roles, and they have thus for some time been the subject of medical research [10], [11]. Anthocyanins have recently been studied for their role of protecting leaf cells from photo-oxidative damage and thereby enhancing the efficiency of nutrient retrieval during senescence [12]. Flavonoids including anthocyanins and non-anthocyanin flavonoids are a remarkably diverse group of secondary products with a vast array of biological functions especially during stress protection [13], [14].

Flavonoids may be analyzed by reversed-phase high performance liquid chromatography (RP-HPLC) coupled with UV-Vis detection and mass spectrometry [15], [16]. Different separation protocols are necessary for the efficient extraction of various target compounds. While some studies have performed a analysis of flavonoids in lotus petals [17], [18], no systemic, comprehensive study involving the simultaneous analysis of anthocyanin and flavonol composition in lotus flower petals seems to be available to date, with the exception of Yang et al. [19]. In that study five anthocyanins and ten flavonols were distinguished, however the separation protocol comprised eight phases and thus is difficult to implement. Our previous study [20] demonstrated that almost all lotus tissues have potential pharmacological value due to the presence of derivatives of the most commonly encountered aglycones, including quercetin, myricetin, kaempferol, isorhamnetin, diosmetin and syringetin.

One important approach to understanding the role of flavonoids in stress responses is to look at biosynthetic pathways, which are still poorly documented in lotuses. An optimized methodology used to detect flavonoids in lotuses across germplasm genotypes and tissues may be an effective way to deduce putative flavonoid biosynthetic pathways based on the identification of flavonoids. Such results would also be useful in research into the molecular regulation of flower color expression and for pharmacological studies. Determination of flavonoid composition and content in various tissues might also be valuable in increasing the bio-availability of lotus products.

Characterization of lotus flavonoid composition and content has mostly been limited to several of the most widely cultivated flower and seed-producing lotus cultivars [19], [21], [22]. Many lotus cultivars can be hybridized for potential germplasm improvement. In this study, flavonoids in nine types of tissue from each of the twelve different lotus cultivars were analyzed with the aim of enabling increased efficiency in the utilization of the various tissues.

## Materials and Methods

### Plant materials

Plants of one rhizome lotus cultivar, six flower lotus cultivars, and three seed lotus cultivars of *N*. *nucifera* together with two cultivars of *N. lutea* were grown at the Wuhan Botanical Garden of the Chinese Academy of Sciences, Wuhan (lat. 30°32′ N long, 116°25′ E, alt.), in separate small pools receiving the same fertilization and disease control. The twelve cultivars were of the five flower petal color types: red, pink, white with red border, white and yellow (Table 1). Three replicates of nine different tissues (leaf pulps, leaf veins, leaf stalks, flower stalks, flower petals, pistils, stamens, fruit coats and seed coats) were manually collected in late July (28°C∼30°C) from three individual plants of each cultivar except the *N. lutea* cultivars, for which no fruit coats or seed coats could be obtained due to abortion. Tissues were immediately frozen in liquid nitrogen, powdered with an analytical mill (IKA A11 basic, IKA) and stored at −40 °C for later analysis.

**Table 1:** Lotus cultivars used in this study.

Genotype groups	Cultivars	Color of flower petal
Rhizome lotus of *N*. *nucifera*	Zhimahuou (1),	Pink
Seed lotus of *N*. *nucifera*	Xianglian (2), Taikong 36 (4),	Pink
	Jianxuan 17 (3),	White with red edge
Flower lotus of *N*. *nucifera*	Honglian (5), Zhuguang (6),	Red
	Ti-1 (8),	Red
	Hongshaoyaolian (7),	Pink
	Ti-3 (9),	White with red edge
	Baige (10),	White
*N. lutea*	Mingmei (11), Jingque (12),	Yellow

### Chemicals

The anthocyanin standards used for quantification were delphinidin 3-*O*-glucoside, cyanidin 3-*O*-glucoside and malvidin 3,5-diglucoside chloride (Sigma). Flavonoid standards [quercetin 3-*O*-rutinoside (rutin), quercetin 3-*O*-galactoside (Qc-3-Gal), quercetin 3-*O*-glucoside (Qc-3-Glu), kaempferol 3-*O*-glucoside (Kae-3-Glu), isorhamnetin 3-*O*-glucoside (Iso-3-Glu)] were obtained from Chromadex. All solvents used for HPLC analysis, including acetonitrile and formic acid, were of HPLC grade (CNW). Other analytical-grade chemicals were obtained from Beijing Chemical Factory.

### Optimization of flavonoid extraction

Two candidate solvent systems of methanol and water (EI; 70∶30 v/v) and methanol, water and formic acid (EII; 70∶28∶2 v/v/v) were used in preliminary tests to optimize the extraction of flavonoids from flower petals. One g of lotus flower petals was extracted with 30 ml EI or EII at 4 °C for 36 hours following protocols detailed in our previous reports [23], [24]; each extract combination was performed in triplicate. The extract was centrifuged for 10 min at 20, 000 g, the supernatant was collected, and the tissues were re-extracted as above twice more. The combined supernatant solutions were evaporated to dryness in a rotary evacuator (RE52AA, Yarong) at 35 °C, then re-dissolved in 2 ml 100% methanol. After purification [20], sample solutions were reconstituted in 4 ml 100% methanol and filtered through a 0.22 µm Millipore filter (Alltech Scientific Corporation) prior to HPLC analysis.

### Qualitative and quantitative analyses of flavonoids

A high performance liquid chromatography (HPLC) unit equipped with a photodiode array detector (Agilent 1290, Agilent) was employed to analyze anthocyanin and non-anthocyanin flavonoids in the tissues. A reverse-phase Waters Sunfire C_18_ column (150 mm×4.6 mm, 3.5 µm) was used for anthocyanin analysis. The mobile phase consisted of 5% formic acid in water as solvent A, and 0.1% formic acid in acetonitrile as solvent B. The gradient profile consisted of 10% B (3 min), 20% B (10 min), 30% B (30 min), and 10% B (5 min), at a flow rate of 0.6 mL/min and a column temperature of 30 °C. The injection volume was 5 µl. Anthocyanin detection in the diode array detector was carried out at 520 nm, and spectrum scans were made from 210 to 600 nm. Non-anthocyanin flavonoid analysis was carried out as per our previous study [24]. All reagents were of HPLC grade. Milli-Q water (Millipore) was used throughout.

Nitrogen was used as the drying agent in the mass spectrometry (MS) analysis. Identification of flavonoids was based on HPLC-UV and HPLC-MS (Agilent 1290 and 6460 triple quadruple mass spectrometry series). Electrospray ionization (ESI) was performed in both positive (PI) and negative (NI) modes for MS analyses. Identification conditions in the positive mode were as follows: HV voltage 3.5 kV; capillary 7 µA; nozzle voltage 500 V; delta EMV 300 V; gas flow 5 L/min; gas temperature 350 °C; nebulizer pressure 45 psi; sheath gas temperature 350 °C; sheath gas flow 11 L/min; scan range 100–1000 m/z units. In negative mode, delta EMV was set at −300 V and scan range at 150–800 m/z units [24]. Additional flavonoid identification was carried out by retention time and UV-Vis spectra obtained by HPLC as well as by comparison to published data.

Quantification of flavonoids with available standards (rutin, quercetin 3-*O*-galactoside, quercetin 3-*O*-glucoside, kaempferol 3-*O*-glucoside, isorhamnetin 3-*O*-glucoside) was performed at 350 nm in the corresponding standard's calibration equation, while the remainders were quantified as rutin. Anthocyanins were quantified at 520 nm as malvidin 3,5-diglucoside chloride equivalents in place of unavailable standards, while delphinidin 3-*O*-glucoside and cyanidin 3-*O*-glucoside were quantified as the corresponding standards.

### Preparation of standard solutions and method validation

The available non-anthocyanin flavonoids standards were accurately weighed and dissolved in 100% HPLC grade methanol. At the same time, anthocyanin standards were dissolved in 2% (v/v) fomic acid-methanol solution. Each standard solution was diluted to a series of concentrations in a wide range to establish calibration curves.

The method was validated by characteristic indexes including linearity, the limit of detection (LOD), the limit of quantification (LOQ) and precision (inter-day, intra-day precision), based on our previous report [20].

### Statistical analysis

Data were analyzed with SPSS 16.0 for Windows. One-way ANOVA with tissues as fixed factors was used to assess the effects of cultivar type (three replicates) on tissue chemical content. Means were tested for significance at the *P*≤0.05 level using a Student-Newman-Keuls test, which was also used to test variation among the tissues. Pearson's correlation coefficient was used to investigate correlation between the two compounds. Principal component analyses (PCA) for identifying homogeneous groups of individual tissues based on measured flavonoid concentrations were performed in SPSS 16.0. Factors were identified using varimax rotation and the two most significant factors were extracted using the Kaiser-Meyer-Olkin criterion (KMO).

## Results

### Anthocyanin extraction

We compared the efficiency of solvent systems EI and EII in extracting anthocyanins from lotus petals. The extraction efficiency of EII was better than that of EI, probably because the solution's acidity affected anthocyanin stability [25]. For this reason EII was used for all anthocyanin extractions. No significant differences between the solvent systems were found for non-anthocyanin flavonoids (*P*≤0.05; data not shown), therefore EI was used for non-anthocyanin flavonoid extraction in a way analogous to that of our previous study [24].

### Anthocyanin analysis

**Figure 1:**
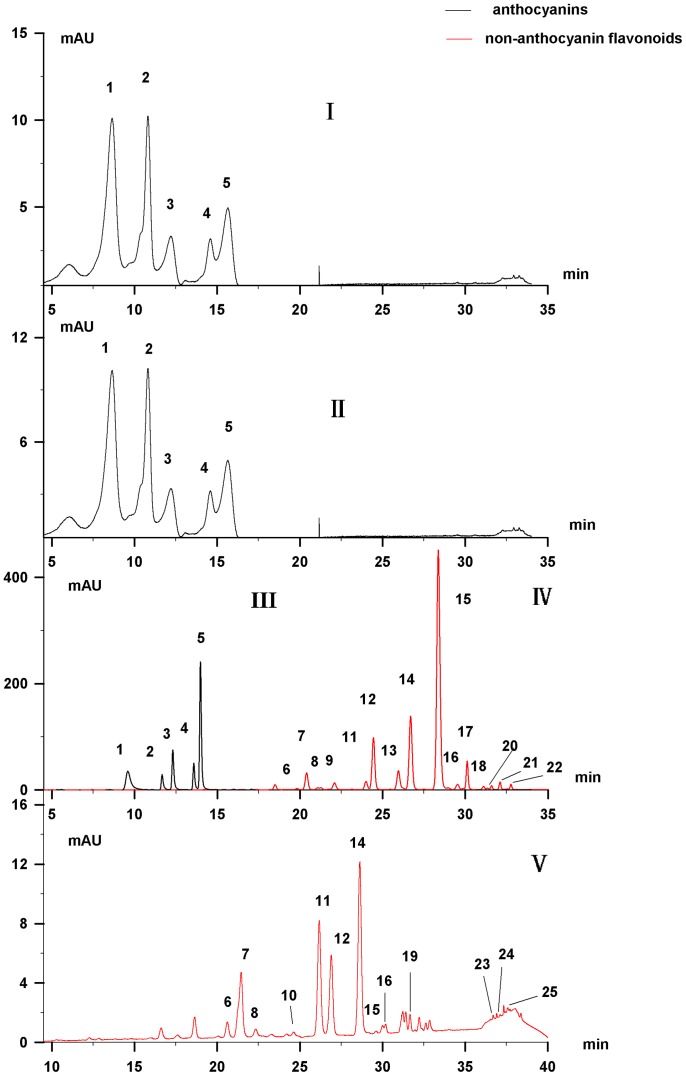
HPLC chromatograms (HPLC-DAD/MS) at 520 nm of anthocyanins (I, II, III) and at 350 nm of non-anthocyanin flavonoids (IV) extracted from flower petals of cultivar ‘Zhuguang’ under an elution system with 0.5% (I, IV), 2% (II) and 5% (III) formic acid. Five anthocyanins (peaks 1–5) were satisfactorily separated with 5% formic acid (III) and twenty non-anthocyanin flavonoids (peaks 6–25) with 0.5% formic acid (IV) in flower petals and stalks. Peak numbers in this figure correspond to compound numbers in Table 2.

The HPLC parameters for separating petal flavonoids were optimized by comparing the performance of several candidate elution systems in identifying fifteen flavonoids from the petals of the cultivar ‘Zhuguang’. The aim of this optimization test was not only to obtain a superior separation efficiency but also to establish an operable system. The SI Elution system, with a mobile phase of 0.5% formic acid (Fig. 1, IV) as described in Chen et al. [24], achieved a fair resolution of non-anthocyanin flavonoids; however, the resolution and peak shape were insufficient for anthocyanins analysis (Fig. 1, I). As the mobile phase's acidity may be an important factor affecting the resolution of flavonoids [20], we tested different mobile phases with the acidity varying from 0.5% to 5%. Results showed that mobile phases with an acidity<2% performed badly in separating the target anthocyanins (Fig. 1, II). A mobile phase of 5% formic acid performed best (Fig. 1, III). This result suggested that a suitable concentration of formic acid as an eluent additive is sufficient for the analysis of anthocyanins and non-anthocyanin flavonoids in lotus flower petals. There appears to be no necessity to add trifluoroacetic acid to the elution system to obtain a satisfactory separation of anthocyanins as reported by Yang et al. [19].

### Identification of flavonoids

After optimized chromatographic conditions, higher separation and peak resolution of target compounds were obtained. All calibration curves showed good linearity (*r*≥0.9986) over a relatively wide concentration range, with over-all the LOD<0.20 µg/ml and LOQ<0.67 µg/ml (LOD estimated as three times the signal of the background noise, while LOQ as 10 times the signal of the background noise) (Table S1). The developed method provided satisfactory precision with inter-day (n = 3) and intra-day (n = 6) variations of 0.02–0.88% and 0.09–3.45%, respectively (Table S2), validating the high repeatability and intermediate precision of the method developed in this study.

The identities of flavonoids (also described in our previous study [24]) were determined from the retention times, molecular ions, fragment ions, and UV-Vis absorbance maxima as revealed by HPLC-MS and HPLC (data not shown). Five anthocyanins (Fig. 1, III) and twenty non-anthocyanin flavonoids (Fig. 1, IV & V) were identified. Peaks 1–5 correspond to the anthocyanins delphinidin 3-*O*-glucoside (Dp-3-Glu, peak 1), cyanidin 3-*O*-glucoside (Cy-3-Glu, peak 2), petunidin 3-*O*-glucoside (Pt-3-Glu, peak 3), peonidin 3-*O*-glucoside (Pn-3-Glu, peak 4), and malvidin 3-*O*-glucoside (Mv-3-Glu, peak 5), as previously reported [19]. Peaks 6–25 correspond to non-anthocyanin flavonoids: 3 myricetin glycosides [myricetin 3-*O*-galactoside (Myr-3-Gal, peak 6), myricetin 3-*O*-glucoside (Myr-3-Glu, peak 7), myricetin 3-*O*-glucuronide (Myr-3-Gln, peak 9)], 6 quercetin glycosides [quercetin 3-*O*-arabinopyranosyl-(1→2)-galactopyranoside (Qc-3-Ara-Gal, peak 8), quercetin 3-*O*-rhamnopyranosyl-(1→6)-glucopyranoside (Qc-3-Rha-Glu/rutin, peak 10), quercetin 3-*O*-galactoside (Qc-3-Gal, peak 11), quercetin 3-*O*-glucoside (Qc-3-Glu, peak 12), quercetin-3-*O*-glucuronide (Qc-3-Gln, peak 14), quercetin (peak 24)], 5 kaempferol glycosides [kaempferol 3-*O*-robinobioside (Kae-3-Rob, peak 13), kaempferol 3-*O*-galactoside (Kae-3-Gal, peak 15), kaempferol 3-*O*-glucoside (Kae-3-Glu, peak 17), kaempferol 3-*O*-glucuronide (Kae-3-Gln, peak 20), kaempferol 7-*O*-glucoside (Kae-7-Glu, peak 21)], 3 isorhamnetines [isorhamnetin 3-*O*-rutinoside (Iso-3-Rut, peak 16), isorhamnetin 3-*O*-glucoside (Iso-3-Glu, peak 19), isorhamnetin 3-*O*-glucuronide (Iso-3-Gln, peak 23)], 2 diosmetin [diosmetin 7-*O*-hexose (Dio-7-Hex, peak 22), diosmetin (peak 25)], and syringetin 3-*O*- glucoside (Syr-3-Glu, peak 18).

### Flavonoid concentrations

Mean contents, content ranges and subtotals of each group for all sampled cultivars are summarized in Table 2. There were non-anthocyanin flavonoids in all tissues while anthocyanins were only detected in flower petals. Flavonoid concentration varied with germplasm and tissue types. Total flavonoid concentration was highest in leaf pulps at 791.82 mg/100 g fresh weight (FW), while the lowest concentrations of<50 mg/100 g FW were found in leaf and flower stalks and seed coats. Next to leaf pulps, petals had significantly higher flavonoid contents (220.1 mg/100 g FW) than the other tissues, and significantly higher flavonoid contents were found in leaf veins and stamens than in pistils and fruit coats.

**Table 2:** Average content of anthocyanins and non-anthocyanin flavonoids (mg/100 g FW) and their distribution ranges (in parentheses) in various tissues of the lotus germplasms studied.

Groups	No.	Compounds	Leaf pulps	Leaf veins	Leaf stalks	Flower stalks	Flower petals	Stamens	Pistils	Seed coats	Fruit coat
Anthocyanins	1	Dp-3-Glu					3.8 (0.0–16.0)				
	2	Cy-3-Glu					1.6 (0.0–4.9)				
	3	Pt-3-Glu					2.9 (0.0–10.1)				
	4	Pn-3-Glu					2.3 (0.0–5.0)				
	5	Mv-3-Glu					10.5 (0.0–31.3)				
		Subtotal					21.4(0.0–67.8)				
Myricetin	6	Myr-3-Gal	1.5b(0.0–2.8)	0.6a(0.0–1.0)	0.4a(0.0–1.5)	0.8a(0.0–2.0)	1.3b(1.0–2.0)	2.1c(1.3–6.5)	1.5b(0.0–4.0)	1.2c(0.0–2.5)	2.1c(0.0–3.9)
	7	Myr-3-Glu	12.9d(2.1–22.3)	3.2ab(1.5–4.9)	1.7a(0.0–2.6)	1.5a(0.4–2.6)	5.9c(2.3–22.7)	6.1c(2.7–11.1)	5.5bc(2.3–17.0)	3.5ab(1.6–4.6)	5.1bc(2.2–9.5)
	9	Myr-3-Gln	5.1b(0.0–12.0)	1.9a(0.0–3.3)	1.0a(0.6–2.4)	1.4a(0.9–2.5)	5.3b(1.0–30.0)	1.8a(0.3–3.6)	8.2c(1.8–21.6)	5.5b(2.0–13.8)	8.7c(4.2–22.6)
		Subtotal	19.5d(6.3–31.5)	5.6ab(2.8–9.4)	3.1a(1.6–5.8)	3.7a(2.4–5.2)	12.5bcd(4.1–54.5)	10.0 abc(4.5–19.7)	15.2cd(4.1–21.0)	10.3abc(4.6–21.3)	15.8cd(6.4–29.6)
Quercetin	8	Qc-3-Ara-Gal	65.5c(41.4–99.9)	13.0b(7.2–21.2)	3.3a(0.9–6.0)	2.1a(0.5–5.0)	2.9a(1.5–4.1)	1.4a(0.0–3.7)	3.0a(1.3–5.8)	1.0a(0.0–1.7)	2.4a(1.5–2.9)
	10	rutin	10.8b(3.9–33.4)	3.1a(0.8–11.2)	1.3a(0.0–3.2)	1.5a(0.0–5.8)	9.5b(1.5–50.7)	11.0b(5.0–26.6)	2.8a(0.9–10.7)	1.2a(0.7–2.2)	1.2a(0.8–2.0)
	11	Qc-3-Gal	110.5c(15.0–207.2)	20.2b(3.0–37.6)	3.4a(1.5–6.1)	5.1a(1.6–9.0)	7.2a(1.9–17.9)	6.3a(3.0–14.0)	4.4a(1.6–8.9)	3.3a(2.4–4.3)	14.8ab(2.5–30.3)
	12	Qc-3-Glu	115.0d(87.4–167.1)	21.0c(11.3–36.5)	5.7a(3.5–8.8)	5.6a(3.4–9.0)	18.4bc(7.9–55.4)	14.4bc(8.2–18.5)	12.3ab(6.5–23.6)	5.7a(3.6–10.1)	12.3ab(10.3–16.4)
	14	Qc-3-Gln	391.7c(228.3–541.8)	80.3b(44.1–154.3)	9.6a(3.1–32.3)	15.9a(5.5–28.0)	61.7b(8.9–266.5)	6.8a(0.7–20.5)	52.1b(9.6–124.7)	17.0a(3.3–51.1)	73.5b(20.8–156.7)
	24	Quercetin	1.4c(0.3–3.1)	0.9ab(0.7–1.2)	1.4c(0.7–2.5)	1.0ab(0.3–1.5)	0.6a(0.0–1.2)	1.1b(0.0–2.3)	1.0ab(0.0–2.1)	0.8ab(0.0–1.3)	0.7c(0.2–1.3)
		Subtotal	695.0d(575.9–871.0)	138.7c(87.2–222.6)	24.7a(13.7–53.2)	31.2a(13.8–45.3)	100.1bc(32.7–396.7)	41.1ab(23.2–75.8)	75.6abc(23.1–150.5)	29.0a(10.8–68.1)	105.1bc(52.3–180.0)
Kaempferol	13	Kae-3-Rob	0.3a(0.0–1.6)	0.1a(0.0–0.9)	0.1a(0.0–0.5)	0.0a(0.0–0.0)	1.0b(0.0–1.8)	1.1b(0.0–1.8)	0.1a(0.0–1.0)	0.0a(0.0–0.0)	0.0a(0.0–0.0)
	15	Kae-3-Gal	10.0b(1.2–23.3)	2.1a(0.9–4.1)	0.9a(0.0–1.4)	1.1a(0.7–1.9)	17.1c(3.4–35.8)	28.4d(4.4–61.6)	2.0a(0.7–3.9)	1.0a(0.0–2.1)	2.3a(1.0–4.0)
	17	Kae-3-Glu	33.8b(5.8–84.7)	3.0a(0.2–6.6)	0.3a(0.1–0.8)	0.5a(0.0–2.5)	39.2b(0.6–70.6)	33.9b(11.4–50.6)	2.0a(0.2–4.4)	0.7a(0.0–1.4)	2.8a(0.5–4.8)
	20	Kae-3-Gln	12.8b(7.4–24.2)	2.4a(1.7–3.9)	0.9ab(0.7–1.2)	1.1a(0.7–1.5)	28.6d(6.1–79.4)	22.8c(5.4–74.7)	2.4a(1.1–4.7)	1.2a(0.8–1.7)	3.3a(1.1–6.0)
	21	Kae-7-Glu	3.3e(0.0–6.0)	1.5c(0.0–2.3)	0.9ab(0.7–1.2)	1.0abc(0.7–1.5)	1.9d(0.0–4.1)	1.4bc(0.0–3.2)	1.3abc(0.0–2.2)	0.8a(0.0–1.3)	2.2d(0.0–5.6)
		Subtotal	60.2b(24.4–128.9)	9.1a(5.1–14.6)	2.9a(1.4–4.6)	3.6a(2.4–6.9)	87.8c(15.5–165.3)	87.6c(36.2–146.2)	7.7a(2.1–12.8)	3.7a(1.1–5.5)	10.6a(6.6–14.4)
Isorhamnetin	16	Iso-3-Rut	1.9a(0.0–8.2)	0.5a(0.0–3.1)	0.4a(0.0–2.0)	0.4a(0.0–2.4)	5.9c(0.0–29.6)	3.9b(0.0–24.2)	0.8a(0.0–5.0)	0.2a(0.0–1.3)	0.2a(0.0–2.0)
	19	Iso-3-Glu	3.7d(0.9–8.9)	0.7ab(0.3–1.6)	0.4a(0.0–1.2)	0.6ab(0.0–1.7)	8.3e(2.2–16.9)	9.1e(1.5–17.1)	2.2bc(0.0–6.2)	1.1ab(0.0–4.0)	3.3cd(1.8–8.9)
	23	Iso-3-Gln	7.1b(0.0–24.6)	2.6a(0.0–3.6)	1.4a(0.0–2.1)	1.7a(0.0–2.9)	1.1a(0.0–2.2)	1.7a(0.0–2.7)	1.3a(0.0–2.1)	1.3a(0.0–2.4)	1.6a(0.7–2.1)
		Subtotal	12.7b(0.9–37.5)	3.8a(0.3–9.6)	2.2a(0.0–3.3)	2.7a(0.2–4.7)	15.4b(3.6–34.6)	14.7b(6.2–31.7)	4.3a(0.7–8.0)	2.5a(0.4–6.2)	5.1a(2.5–11.0)
Diosmetin	22	Dio-7-Hex	2.8e(1.7–4.4)	1.3bc(0.8–2.3)	0.7ab(0.0–1.2)	1.0b(0.0–1.6)	1.6c(0.0–4.3)	1.2bc(0.0–2.7)	1.2bc(0.6–2.3)	0.5a(0.0–1.3)	2.2d(1.1–6.2)
	25	Diosmetin	1.5a(0.0–2.9)	1.2a(0.0–1.9)	1.0a(0.0–2.1)	0.9a(0.0–3.1)	1.3a(0.0–3.3)	1.6a(0.0–5.5)	1.5a(0.0–3.1)	1.5a(0.0–3.8)	1.1a(0.1–3.1)
		Subtotal	4.2b(2.0–7.1)	2.5a(1.4–3.3)	1.7a(0.7–3.1)	1.9a(1.0–4.4)	2.9ab(0.0–5.8)	2.8ab(0.0–6.8)	2.8ab(0.7–4.5)	2.0a(0.0–4.7)	3.2ab(1.2–6.9)
Syringetin	18	Syr-3-Glu	0.2a(0.0–2.5)	0.2a(0.0–1.1)	0.2a(0.0–1.0)	0.2a(0.0–1.1)	1.3ab(0.0–6.5)	1.4ab(0.0–8.9)	1.9b(0.0–5.7)	1.2ab(0.0–3.3)	2.3b(0.0–5.5)
		Total	791.8e(642.6–1050.1)	159.9c(106.5–247.3)	34.7a(21.7–68.1)	43.3a(23.1–61.7)	220.1d(120.1–467.2)	157.6c(108.3–247.1)	107.5b(41.9–188.2)	48.7a(18.0–104.5)	142.1bc(97.3–219.3)

Letters indicate significant differences between tissues at *P*<0.05.

Notes: Dp-3-Glu = delphinidin 3-*O*-glucoside; Cy-3-Glu = cyanidin 3-*O*-glucoside; Pt-3-Glu = petunidin 3-*O*-glucoside; Pn-3-Glu = peonidin 3-*O*-glucoside; Mv-3-Glu = malvidin 3-*O*-glucoside; Myr-3-Gal = myricetin 3-*O*-galactoside; Myr-3-Glu = myricetin-3-*O*-glucoside; Myr-3-Gln = myricetin 3-*O*-glucuronide; Qc-3-Ara-Gal = quercetin 3-*O*-arabinopyranosyl-(1→2)-galactopyranoside; Qc-3-Gal = quercetin 3-*O*-galactoside; Qc-3-Glu = quercetin 3-*O*-glucoside; Qc-3-Gln = quercetin-3-*O*-glucuronide; Kae-3-Rob = kaempferol 3-*O*-robinobioside; Kae-3-Gal = kaempferol 3-*O*-galactoside; Kae-3-Glu = kaempferol 3-*O*-glucoside; Kae-3-Gln = kaempferol 3-*O*-glucuronide; Kae-7-Glu = kaempferol 7-*O*-glucoside; Iso-3-Rut = isorhamnetin 3-*O*-rutinoside; Iso-3-Glu = isorhamnetin 3-*O*-glucoside; Iso-3-Gln = isorhamnetin 3-*O*-glucuronide; Dio-7-Hex = diosmetin 7-*O*-hexose; Syr-3-Glu = syringetin 3-*O*- glucoside.

Content of compounds 1, 2, 10, 11, 12, 17, and 19 were quantified by comparison with external standards, while compounds 3, 4, 5 are given in mg/100 g FW equivalent of malvidin 3,5-diglucoside chloride. The other non-anthocyanin flavonoids were quantified as rutin.

### Leaf pulps and veins

The greatest abundance of flavonoids was found in leaf pulps, with a mean total flavonoid content 3–22 times higher than in the other tissues (Table 2). Among germplasms, the seed lotus ‘Taikong 36’ had the highest flavonoid content (1050.1 mg/100 g FW) among the 12 cultivars studied. The *N. lutea* cultivar ‘Jingque’ and seed lotus ‘Jianxuan 17’ had total leaf pulp flavonoid contents of 933.4 mg/100 g FW and 919 mg/100 g FW, respectively, significantly higher than the remaining cultivars. ‘Ti-3’ had the lowest flavonoid content at only 642.6 mg/100 g FW. Flavonoids in leaf veins were significantly less concentrated than in leaf pulps, ranging from 106.5–247.3 mg/100 g FW, with a mean content of 159.9 mg/100 g FW. Two cultivars, *N. lutea* cultivar ‘Jingque’ and the rhizome lotus ‘Zhimahuou’, had significantly higher leaf vein flavonoid contents than the others.

**Figure 2:**
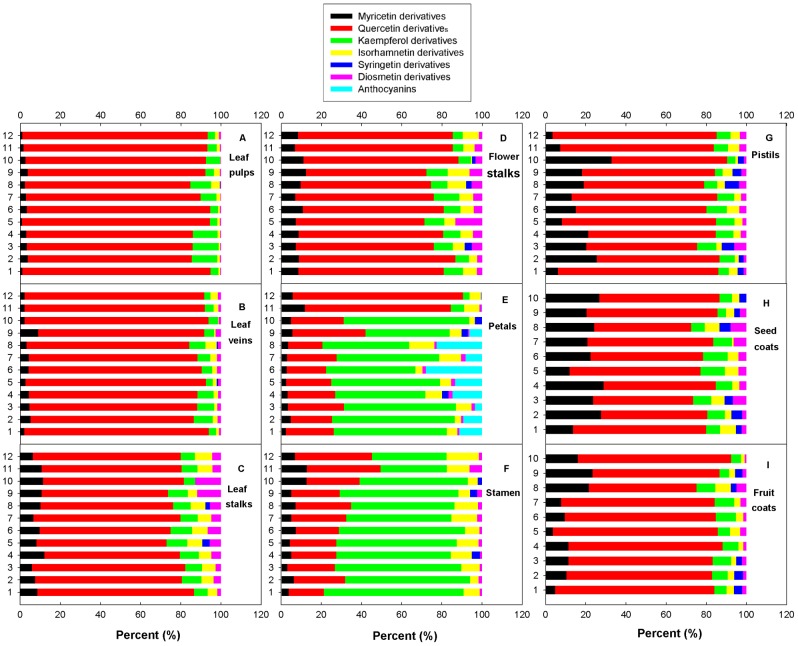
Percentages contributed by the group of flavonoid derivatives to total flavonoids in various tissues of the different genotypes. Cultivar designations (1–12; Y-axis) correspond to those in Table 1.

Among the six groups of individual glycosides, quercetin glycosides were the dominant flavonoids detected in leaf pulps and veins, accounting for more than 80% of the total in all studied cultivars (Fig. 2 A, B). Three quercetin glycosides, Qc-3-Gal (11), Qc-3-Glu (12) and Qc-3-Gln (14), were the most abundant monomeric glycosides in leaf pulps and veins, making up >66.5% of total detected flavonoids (Table 2). Regarding the other free forms of flavonoids, the dihydroxylated flavonoid Qc-3-Ara-Gal (8) was also relatively abundant, accounting for 11.5% and 12.6% of total in leaf pulps and veins respectively.

### Leaf and flower stalks

Leaf and flower stalks were the two tissues with the lowest flavonoid concentrations, with respective averages of 34.7 and 43.3 mg/100 g FW (Table 2). The rhizome lotus ‘Zhimahuou’ had the highest content of flavonoids in leaf and flower stalks by a significant margin. Three flower lotus cultivars, ‘Hongshaoyaolian’ (42.2 mg/100 g FW), ‘Ti-1’ (40.5 mg/100 g FW) and ‘Mingmei’ (41.1 mg/100 g FW), had relatively higher total flavonoid contents in leaf stalks. For flower stalk flavonoid concentrations, the seed lotus ‘Xianglian’ (57.8 mg/100 g FW) and *N. lutea* cultivar ‘Jingque’ (58.8 mg/100 g FW) surpassed ‘Zhimahuou’. The lowest flavonoid contents were detected in leaf and flower stalks of the cultivar ‘Ti-3’.

As with the leaf pulps and veins, quercetin glycosides also made up the biggest percentage of flavonoids in leaf and flower stalks, accounting for 59.9%–78.6% among the cultivars (Fig. 2 C, D). However, kaempferol glycosides and myricetin glycosides made up a relatively large proportion of the flavonoids in leaf and flower stalks (4.9%–12.8% and 5.9%–12.3% respectively). Diosmetin glycosides accounted for no more than 3% of the total in leaf pulps and veins, but were more concentrated in the leaf and flower stalks of some cultivars; for example they made up >10% in leaf stalks of ‘Ti-1’ (11.8%) and ‘Baige’ (12.8%) and in flower stalks of ‘Honglian’ (13.3%). Among individual flavonoids, quercetin glycosides Qc-3-Gal (11), Qc-3-Glu (12) and Qc-3-Gln (14) were abundant, making up 40.7%–67.8% of the total in all cultivars (Table 2). In the leaf and flower stalks of the rhizome cultivar ‘Zhimahuou’, the three quercetin monomers accounted respectively for 67.8% and 60.1% of all flavonoids (Fig. 2 C, D). In contrast, aglycone quercetin (24) and Iso-3-Gln (23) concentrations were higher than in leaf pulps and veins (Table 2).

### Flower petals

In lotuses, anthocyanins are found located in the flower petals. The content of flavonoids was seen to be relatively high, ranging from 120.1 to 467.2 mg/100 g FW, significantly higher than in other tissues excluding leaf pulp (Table 2). Flavonoid content in petals strongly depended on genetic background. The yellow petal cultivars ‘Jingque’ (459.1 mg/100 g FW) and ‘Mingmei’ (467.2 mg/100 g FW) of *N. lutea* had the highest total flavonoids, significantly higher than all cultivars of *N*. *nucifera*. Within *N*. *nucifera*, a relatively high flavonoid content was found in ‘Honglian’, ‘Zhuguang’, ‘Zhimahuou’, ‘Xianglian’ and ‘Ti-3’, with concentrations between 200 and 300 mg/100 g FW. The remainder of the cultivars had concentrations of ∼150 mg/100 g FW.

The composition of flavonoids in the petals of *N. lutea* differed from that of *N*. *nucifera* (Fig. 2 E). *N. lutea* had almost no anthocyanins and was also characterized by a relatively high concentration of quercetin derivatives (>72.7 % of total flavonoids). In contrast, *N*. *nucifera* flower petals contained anthocyanins [except for the white petal cultivar ‘Baige’ (10)] and showed higher levels of kaempferol derivatives (41.9%–62.6% of total flavonoids) and quercetin derivatives (∼23% of total flavonoids on average). Relatively high levels of anthocyanin were found in red-petaled cultivars ‘Zhuguang’ (27.9% of total flavonoids) and ‘Ti-1’ (22.6%), followed by the pink-petaled cultivar ‘Taikong 36’ (14.7%) and the red-petaled cultivar ‘Honglian’ (13.4%).

Among individual flavonoids, quercetin monomers Qc-3-Glu (12) and Qc-3-Gln (14) were the dominant glycosides in *N. lutea*, accounting respectively for 61.4% and 69.0% of total glycosides in the petals of ‘Jingque’ and ‘Mingmei’. However, three major kaempferol glycosides (Kae-3-Gal, 15; Kae-3-Glu, 17; Kae-3-Gln, 20) were abundant in *N*. *nucifera* and made up >42.9% of the total in all cultivars. Additionally a total of five anthocyanins were detected in petals. Mv-3-Glu (5) was the dominant anthocyanin compound in petals and made up >42.3% of anthocyanins across all cultivars. The other four anthocyanin derivatives were present in nearly equal amounts.

### Flower stamens

Total flavonoid content in flower stamens did not vary significantly among cultivars, ranging from 108.3 to 247.1 mg/100 g FW (Table 2). The highest concentration was detected in ‘Ti-1’ (247.1 mg/100 g FW), followed by ‘Mingmei’ (197.3 mg/100 g FW) and ‘Honglian’ (192.8 mg/100 g FW). The lowest was found in the red flower lotus ‘Zhuguang’ (108.3 mg/100 g FW).

**Figure 3:**
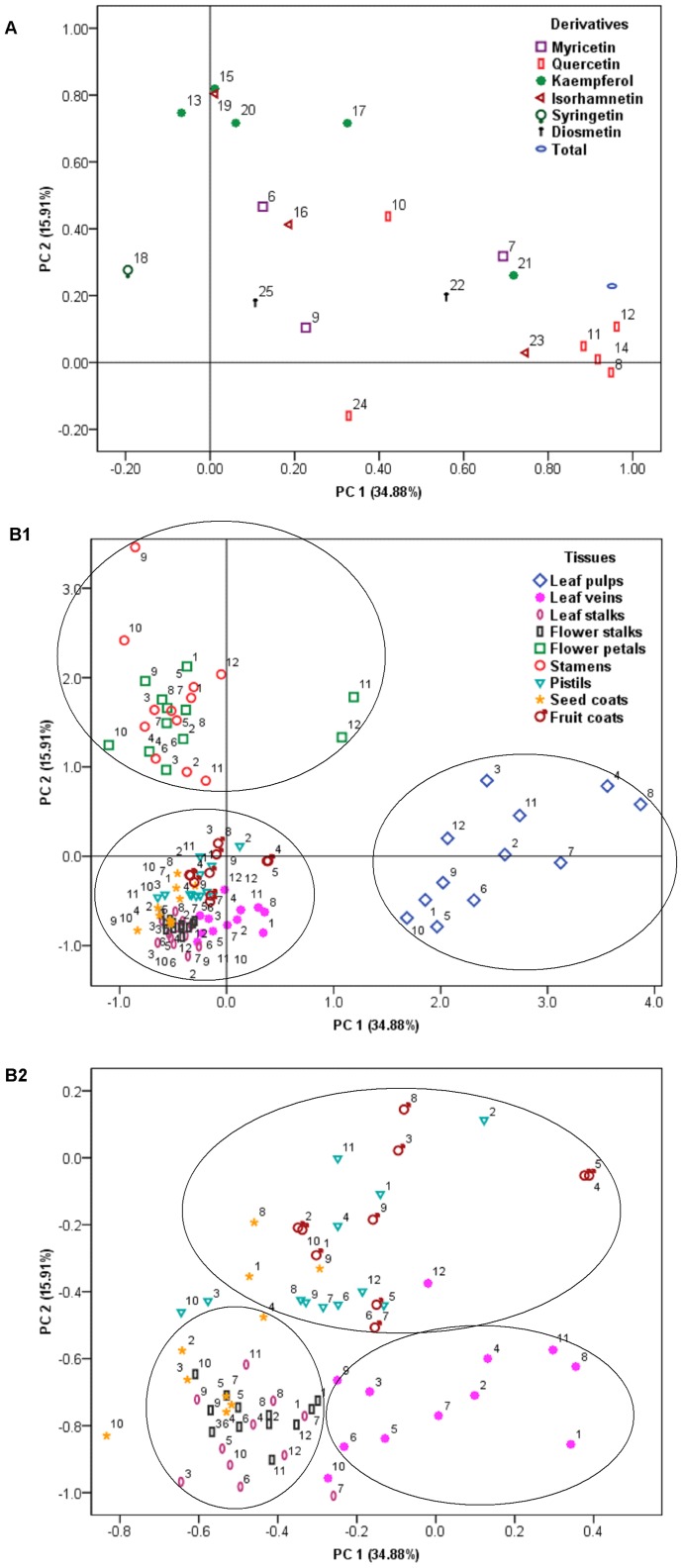
Positions of PCA scores (PC1, PC2) of nine lotus tissues, based on non-anthocyanin flavonoid compound content. Percentages in parentheses represent principal component variance. Flavonoid numbers in figure A correspond to those in Table 2 and numbers in figures B1 and B2 correspond to cultivar numbers in Table 1.

Kaempferol glycosides and quercetin derivatives were seen to be two important flavonoids in flower stamens. In contrast to flower petals, there were far fewer differences in flavonoid types between *N*. *nucifera* and *N. lutea* (Fig. 2 F). Kaempferol glycoside accounted for >50% of total flavonoids in all *N.nucifera* cultivars and made up ∼30% of total flavonoids in flower stamens of the two *N. lutea* cultivars. The quercetin content was similar among the two species at ∼35% of total flavonoids. There were, however, great differences in individual flavonoids between the two species (Fig. 3 F). Three dominant compounds, Kae-3-Gal (15), Kae-3-Glu (17) and Kae-3-Gln (20), accounted for more than 49.5% in all *N*. *nucifera* cultivars, while non-predominant compounds were found in two *N. lutea* cultivars. Myr-3-Glu (7), rutin (10), Qc-3-Glu (12), Qc-3-Gln (14), Kae-3-Gal (15), Kae-3-Glu (17), Kae-3-Gln (20) and Iso-3-Rut (16) each accounted for about 10% of total flavonoids in *N. lutea*.

### Flower pistils

Total flavonoid content was lower in flower pistils than in flower petals and stamens, ranging from 41.9 to 188.2 mg/100 g FW (Table 2). Quercetin glycosides were abundant in flower pistils and accounted for 55.2% (‘Taikong 17’) to 81.7% (‘Mingmei’) of total flavonoids (Fig. 2 G). Myricetin glycosides were the second most abundant flavonoids at ∼15% of the total in most cultivars. Qc-3-Gln (14) was a predominant flavonoid in most cultivars except ‘Jianxuan 17’ and ‘Baige’ (9.8 and 16.3 mg/100 g FW). Together with two other quercetin glycosides, Qc-3-Gal (11) and Qc-3-Glu (12), these compounds accounted for 48.7% in ‘Ti-1’ and >60% of total flavonoids in the other cultivars.

### Fruit and seed coats

Average total flavonoid content in fruit coats was nearly equal to that in fruit stamens at 142.2 mg/100 g FW (Table 2), but seed coats had significantly lower flavonoid content than fruit coats, with an average of 48.7 mg/100 g FW. However, derivative levels in fruit coats were similar to those in seed coats. Quercetin derivatives were predominant compounds in both fruit and seed coats (Fig. 2 I, H). Myricetin derivatives had an important role, making up >20% in all seed coats except in ‘Zhimuhuou’ (13.6%) and ‘Honglian’ (11.9%). The quercetin compounds Qc-3-Gal (11) and Qc-3-Gln (14) were most abundant in fruit coats, accounting for >48.7% of total flavonoids, while myricetin glycoside Myr-3-Gal (6) was generally significantly higher in seed coats than in fruit coats. Myr-3-Gal (6), Qc-3-Gal (11), Qc-3-Glu (12) and Qc-3-Gln (14) were abundant in seed coats in all cultivars, accounting for >48.2% of total flavonoids.

### Correlations among flavonoid compounds

Anthocyanins and non-anthocyanin flavonoids were detected in petals while in the other tissues except petals were only found non-anthocyanin flavonoids. It is therefore instructive to generate correlation coefficients among different groups of flavonoid compounds separately for flower petals and the other tissues (Table 3).

**Table 3:** Correlation coefficients of each flavonoid derivative in petals of all studied cultivars (above diagonal) and of each flavonoid derivative in other tissues (below diagonal).

	Dp	Cy	Pt	Pn	Mv	Myr	Qc	Kae	Iso	Syr	Dio	Total
Dp	**1.00**	0.95**	0.96**	0.85**	0.91**	−0.36	−0.39	0.45	−0.29	−0.25	0.37	−0.31
Cy	-	**1.00**	0.91**	0.85**	0.88**	−0.47	−0.52	0.59*	−0.36	−0.27	0.36	−0.42
Pt	-	-	**1.00**	0.95**	0.98**	−0.41	−0.43	0.53	−0.21	−0.25	0.39	−0.33
Pn	-	-	-	**1.00**	0.98**	−0.49	−0.51	0.63*	−0.16	−0.21	0.33	−0.39
Mv	-	-	-	-	**1.00**	−0.43	−0.45	0.59*	−0.18	−0.25	0.34	−0.33
Myr	-	-	-	-	-	**1.00**	0.86**	−0.59*	0.77**	−0.17	0.39	0.87**
Qc	-	-	-	-	-	0.57**	**1.00**	−0.67*	0.75**	−0.23	0.18	0.96**
Kae	-	-	-	-	-	0.45**	0.37**	**1.00**	−0.54	−0.11	0.13	−0.46
Iso	-	-	-	-	-	0.35**	0.40**	0.68**	**1.00**	−0.36	0.44	0.76**
Syr	-	-	-	-	-	0.24*	−0.12	0.21	0.12	**1.00**	−0.53	−0.31
Dio	-	-	-	-	-	0.35**	0.55**	0.32**	0.40**	0.03	**1.00**	0.31
Total	-	-	-	-	-	0.62**	0.99**	0.51**	0.50**	−0.06	0.57**	**1.00**

The symbols * and ** indicate significant correlations at P <0.05 and P <0.01 respectively.

Notes: Dp = delphinidin 3-*O*-glucoside; Cy = cyanidin 3-*O*-glucoside; Pt = petunidin 3-*O*-glucoside; Pn = peonidin 3-*O*-glucoside; Mv = malvidin 3-*O*-glucoside; Myr = myricetin derivatives; Qc = quercetin derivatives; Kae = kaempferol derivatives; Iso = isorhamnetin derivatives; Syr = syringetin 3-*O*-glucoside; Dio = diosmetin derivatives.

Correlation coefficients among the five anthocyanins in petals were very high (0.85–0.98) (Table 3, above diagonal). Regarding the relationship between anthocyanin and non-anthocyanin flavonoids, high correlation coefficients (0.45–0.63) were found only between all five anthocyanins and the kaempferol derivatives. Kaempferol derivatives were also significantly negatively correlated with quercetin; myricetin and isorhamnetin derivatives positively correlated with quercetin derivatives. The total flavonoid content of all studied lotuses were positively correlated with quercetin, myricetin and isohamnetin derivatives, suggesting that variation in these three groups of compounds might dramatically affect total compound content.

Relationships between non-anthocyanin flavonoids in the other tissues except flower petals, in which anthocyanin synthesis did not occur, are listed in Table 3 (below diagonal). Interestingly, the negative correlations between the kaempferol derivatives and quercetin, and the kaempferol and myricetin derivatives were not apparent and were replaced by positive correlations (some significant) in the other tissues (r = 0.21–0.68). The myricetin derivatives showed positive and significantly positive correlations with the other derivatives (r = 0.24; r≥0.35). Finally, nearly all the groups of compounds except for the syringetin derivatives showed positive correlations with total flavonoid content in tissues in which anthocyanin synthesis did not occur.

### Principal Component Analysis (PCA)

The PCA results provide an overview of the complete data set, showing variability between tissues and their flavonoid compounds. PCA on these attributes could explain 50.79% of the variance, partitioned as 34.88% in principal component 1 (PC1) and 15.91% in PC2 (Fig. 3). The loading of both principal components showed strong positive correlations–four quercetin derivatives [Qc-3-Ara-Gal (8), Qc-3-Gal (11), Qc-3-Glu (12), Qc-3-Gln 14)] for PC1, and four kaempferol derivatives [Kae-3-Rob (13), Kae-3-Gal (15), Kae-3-Glu (17), Kae-3-Gln (20)] and one isorhamnetin [(Iso-3-Glu (18)] for PC2. Additionally, Kae-7-Glu (21), Myr-3-Glu (7) and Iso-3-Gln (23) as well as the total flavonoid content showed an important positive correlation with the loadings of PC1. In combination, the two parts of Fig. 3 show the relationships between lotus tissues and their flavonoid compounds. As shown in Fig. 3 B1, lotus tissues can be divided into three groups based on their position in the PCA score scatter plot.

Leaf pulps of all cultivars were clustered on the right with high positive PC1 values and characterized by high content of quercetin derivatives [Qc-3-Glu (12), Qc-3-Gal (11), Qc-3-Gln (14), Qc-3-Ara-Gal (8)] and high total flavonoid content. Flower petals and stamens were clustered together in the upper part of the PC 2 axis, which could be explained by the presence of a high level of kaempferol derivatives [Kae-3-Gal (15), Kae-3-Glu (17), Kae-3-Gln (20), Kae-3-Rob (13)] and Iso-3-Glu (19). The other tissues, which were clustered around the origin, were characterized by relatively low flavonoid content. They could be classified into three sub-groups (Fig. 3, B2). Leaf veins were clustered by positive PC1 values based on relatively high quercetin derivative content. Fruit coats and pistils were characterized by a higher content of kaempferol derivatives and Iso-3-Glu. Flower stalks, leaf stalks and seed coats had low flavonoid levels with no compound dominating.

**Figure 4:**
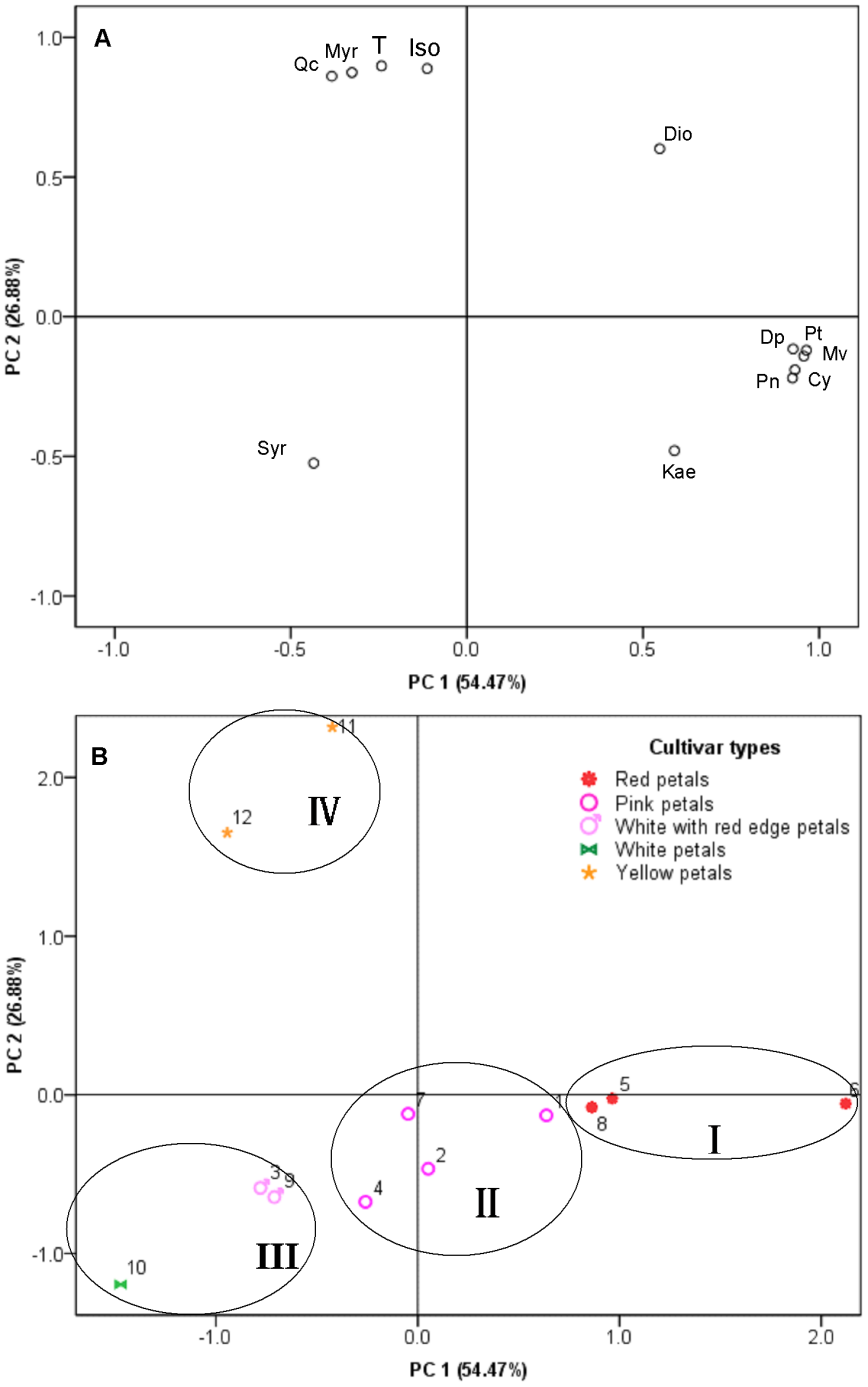
PCA analysis (PC1, PC2) of flavonoids in twelve lotus cultivars based on flavonoid content including anthocyanin and non-anthocyanin flavonoid compounds in flower petals. (A) Score scatter plot; (B) loading plot. Percentages in parentheses represent principal component variance. Numbers in Figure B represent the accession number, corresponding to the numbers in Table 1. Dp = delphinidin 3-*O*-glucoside; Cy = cyanidin 3-*O*-glucoside; Pt = petunidin 3-*O*-glucoside; Pn = peonidin 3-*O*-glucoside; Mv = malvidin 3-*O*-glucoside; Myr = myricetin derivatives; Qc = quercetin derivatives; Kae = kaempferol derivatives; Iso = isorhamnetin derivatives; Syr = syringetin 3-*O*-glucoside; Dio = diosmetin derivatives T = total flavonoid contents.

PCA was also performed on the detected flavonoids including the anthocyanins and non-anthocyanin flavonoids in the petals of the different cultivars (Fig. 4). The scatter plot of score of the first two principal components (PC1 54.47% and PC2 26.88%) was 81.35%, which was high enough to represent all the variables.

All lotus cultivars could be divided into four groups. Group I, formed by the red flower cultivars ‘Zhuguang’ (6), ‘Honglian’ (5) and ‘Ti-1’ (8), was located to the right of the PC1 axis. These three red-petaled cultivars were characterized by high contents of anthocyanins. Group II consisted of four pink flower cultivars (‘Zhimahuoulian’ (1), ‘Xianglian’ (2), ‘Taikong 36’ (4), ‘Hongshaoyaolian’ (7)) characterized by lower levels of anthocyanins than group I, and higher contents of kaempferol glycosides than the other three groups. Group III was located in the third quadrant and contained the red-bordered flower lotuses ‘Ti-3’ and ‘Jianxuan 17’ and the white flower cultivar ‘Baige’. They were characterized by relatively high levels of syringetin derivatives and meager or undetectable levels of anthocyanins. Group IV, which included the two yellow *N*. *lutea* cultivars ‘Jingque’ and ‘Mingmei’, was located in the second quadrant due to the high levels of quercetin, myricetin, isorhamnetin derivatives and low levels of anthocyanins.

## Discussion

In this survey, we reported a flavonoid profile study on various tissues of lotus which widely growing in East Asia, Australia and North America. At the same time, this study aims to revalue the herbs because they are an extraordinary source of antioxidants even through their different tissues have been neglected. The increasing demand for natural antioxidants (functional food additions) justifies the search for new sources of natural antioxidants. The revaluation of various tissues of lotus will be interesting for possibility of producing new commercial products, such as functional food supplements of high quality as well as low costs, furthermore, various lotus tissues including seed coats, leaf and flower stalks which are commonly discarded could be developed for deep pharmacological application.

**Figure 5:**
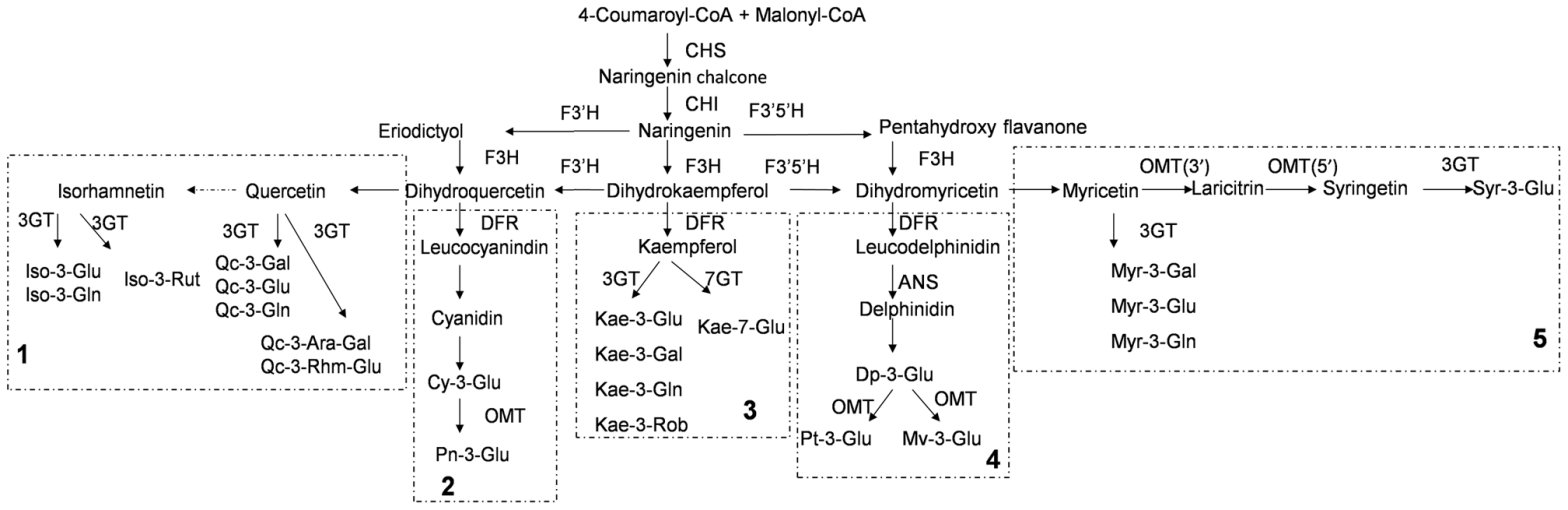
Putative flavonoid biosynthesis pathway in lotus. Notes: Dp: delphinidin; Cy: cyanidin; Pt: petunidin; Pn: peonidin; Mv: malvidin; Myr: myricetin; Qc: quercetin; Kae: kaempferol; Iso: isorhamnetin; Syr: syringetin; Glu: glucoside; Gal: galactoside; Gln: glucuronide; Rob: robinobioside; CHS: chalcone synthase; CHI: chalcone isomerase; F3H: flavonoid 3-hydroxylase; F3′H: flavonoid 3′-hydroxylase; F3′5′H: flavonoid 3′,5′-hydroxylase; DFR: dihydroflavonol reductase; ANS: anthocyanidin synthase; FLS: flavonol synthase; LDOX: leucoanthocyanidin dioxygenase; GT: flavonoid glycosyltransferase; OMT: *O*-methyltransferase; CoA: acethl coenzyme A.

Biosynthetic pathways leading to specific compounds may be deduced by metabolomic analysis across yield from different genotypes [26]. In lotuses these pathways have not yet been detailed, although different kinds of flavonoids have been detected. A putative lotus flavonoid biosynthesis pathway can be deduced for the anthocyanin and non-anthocyanin flavonoids detected in this study (Fig. 5). It is similar to the flavonoid biosynthetic pathway, which is one of the most extensively studied pathways in some plants [12]. It employs Coumaroyl-CoA and Malonyl-CoA and synthesizes dihydrokaempferol with the help of chalcone synthase (CHS), chalcone isomerase (CHI) and flavonoid 3-hydroxylase (F3H). The pathway then divides into five sub-pathways for the synthesis of the anthocyanin and non-anthocyanin flavonoids. It was possible to infer, from their functions, which enzymes [such as flavonoid 3′-hydroxylase (F3′H), flavonoid 3′,5′-hydroxylase (F3′5′H) and dihydroflavonol reductase (DFR)] were responsible for the biosynthesis of various flavonoid detected [27]. Hydroxylation of flavonoids is completed by the action of different enzymes producing aglycones of the favonoids. Finally the flavonoids, with the assistance of the enzyme OMT and others, are glycosylated by glycosyltransferase at a different linkage position.

Our study shows that 3-*O*-glycosylation was the most common characteristic of the anthocyanin and non-anthocyanin lotus flavonoids (Table 2). Glycosylation patterns in lotuses appear to be much simpler than in other plants and generally affected the 3-*O*-position, with the exception of kaempferol and diosmetin–for these substances, two compounds with glycosylation in the 7-*O*-position (Kae-7-Glu and Dio-7-Hex) were detected. Glycosylation in lotuses shows a preference for glucose substituents. Glucuronides are a very rare group of kaempferol glycosides in other plants [28] although kaempferol 3-*O*-glucuronide represented an important compound especially in the stamens and petals of the Chinese lotus (Fig. 3). Methylation and acylation at positions other than the glycosylation position may depend upon the function of the glycosyltransferase during environmental and developmental changes. The function of different enzymes, which may have an effect on flavonoid concentration via the flavonoid synthesis pathway, still needs to be further studied.

In anthocyanin-synthesizing tissues there were significantly positive correlations between anthocyanins and kaempferol derivatives (Table 3), validating the conclusion that sub-pathways 2, 3 and 4 were coordinated in petals (Fig 5). Negative correlations between kaempferol derivatives and quercetin as well as myricetin in petals were observed; these were, however, offset by significantly positive correlations in the other (no anthocyanin-synthesizing) tissues (Table 3). The results show that sub-pathway 3 restricted sub-pathways 1 and 5 if there was anthocyanin synthesis occurring. In non-anthocyanin flavonoid synthesizing tissues, sub-pathways 2 and 4 were not present, while only sub-pathways 1, 3 and 5 were active and coordinated, as validated by significantly positive correlations between non-anthocyanin flavonoids in these tissues. Additionally, the isorhamnetin derivatives showed significant positive correlations with the quercetin derivatives as they were both part of sub-pathway 1.

The use of metabolic analysis to distinguish between some cultivars has been employed in other research. For example, flavonols and polyphenolic profiles have recently been used in chemometrics and were shown to be effective in discriminating between cultivars [29]–[31]. In this study, we demonstrate that petal color in lotuses is also related to patterns of anthocyanin acylation and non-anthocyanin flavonoid types. This pathway, possibly including specific sub-pathways in different cultivars, can synthesize different derivatives. High contents of kaempferol glycosides accompanying anthocyanins were detected in the petals of *N. nucifera*, while high quercetin glycoside concentrations occurred in *N. lutea* (Fig. 2 and 4), supporting the apparent differences in flavonoid synthesis pathways between the two species. Such inter-species differences likely indicate that sub-pathways 2, 3 and 4 are coordinated in lotuses (*N. nucifera*), and that the synthesis of flavonoids is much more effective through sub-pathway 1 if sub-pathways 2, 3 and 4 are blocked (*N. lutea*). The white cultivar ‘Baige’ and red-bordered cultivar ‘Ti-3’ had high contents of syringetin derivatives (Fig. 4). We therefore suggest that sub-pathways 2 and 4 may have an effect on sub-pathway 5; the synthesis of flavonoids shifts to the more effective sub-pathway 5 when a reduction in anthocyanin synthesis occurs.

The PCA results show that the synthesis pathway depended upon the hereditary characterization of tissues. We also demonstrate that metabolic compounds, accumulating in various tissues, can be effective in tissue classification. The use of flavonoids as a means of discrimination is particularly appealing in tissues where bioavailability is of interest, in which case a classification based on anthocyanin and non-anthocyanin flavonoids is possible.

## Supporting Information

Table S1 **Linearity, LOD and LOQ in the determination of three anthocyanins and five non-anthocyanin flavonoid standards.**
(DOC)

Table S2 **Intra- and inter-day precision for each of the lotus petals flavonoids separated by HPLC.**
(DOC)
