# Supplementary material for: Simultaneous Analysis of Anthocyanin and Non-Anthocyanin Flavonoid in Various Tissues of Different Lotus (Nelumbo) Cultivars by HPLC-DAD-ESI-MSn
Source: PLoS One. 2013 Apr 30;8(4):e62291. doi: 10.1371/journal.pone.0062291 (PMC3640015; doi:10.1371/journal.pone.0062291)
Supplement: Table S1 — Linearity, LOD and LOQ in the determination of three anthocyanins and five non-anthocyanin flavonoid standards. (DOC) [file pone.0062291.s001.doc]

**Table S1**

Linearity, LOD and LOQ in the determination of three anthocyanins and five non-anthocyanin flavonoid standards

| Groups | Compounds | Regression equation | *r*^2^ | Linear range | LOD | LOQ |
| --- | --- | --- | --- | --- | --- | --- |
|  |  |  |  | (μg/mL) | (μg/mL) | (μg/mL) |
| Anthocyanins | Malvidin 3,5-diglucoside chloride | *y* = 23.13*x* − 24.95 | 0.9999 | 4.01 − 128.33 | 0.11 | 0.38 |
|  | Delphinidin 3-O-glucoside | *y* = 17.24*x* − 18.51 | 0.9999 | 3.07 − 98.33 | 0.14 | 0.47 |
|  | Cyanidin 3-O-glucoside | *y* = 25.69*x* − 25.47 | 0.9999 | 3.15 − 100.3 | 0.13 | 0.35 |
| Non-anthocyanins | Rutin | *y* = 25.65*x* *−* 29.18 | 0.9992 | 9.38 *−* 250.00 | 0.20 | 0.67 |
| flavonoids | Quercetin 3-*O*-galactoside | *y* = 40.57*x* *−* 100.23 | 0.9994 | 5.73 *−* 183.33 | 0.10 | 0.33 |
|  | Quercetin 3-*O*-glucoside | *y* = 39.28*x* *−* 135.00 | 0.9994 | 7.50 *−* 240.00 | 0.07 | 0.25 |
|  | Kaempferol 3-*O*-glucoside | *y* = 30.21*x* + 18.85 | 0.9994 | 3.13 *−* 100.00 | 0.16 | 0.52 |
|  | Isorhamnetin 3-*O*-glucoside | *y* = 23.46*x* + 3.87 | 0.9986 | 4.69 *−* 150.00 | 0.12 | 0.40 |

Note: *y*, peak area; *x*, compound concentration (μg/mL); LOD = limit of detection, S/N=3; LOQ = limit of quantitation, S/N=10
