# Supplementary material for: Simultaneous Analysis of Anthocyanin and Non-Anthocyanin Flavonoid in Various Tissues of Different Lotus (Nelumbo) Cultivars by HPLC-DAD-ESI-MSn
Source: PLoS One. 2013 Apr 30;8(4):e62291. doi: 10.1371/journal.pone.0062291 (PMC3640015; doi:10.1371/journal.pone.0062291)
Supplement: Table S2 — Intra- and inter-day precision for each of the lotus petals flavonoids separated by HPLC. (DOC) [file pone.0062291.s002.doc]

**Table 2**

Intra- and inter-day precision for each of the lotus petals flavonoids separated by HPLC

| No^1^ | Compounds | Inter-day (n=3) | |  | Intra-day (n=6) | |
| --- | --- | --- | --- | --- | --- | --- |
|  |  | Concentration  (mg/100 g)^2^ | RSD^3^ (%) |  | Concentration  (mg/100 g) | RSD (%) |
| 1 | delphinidin 3-*O*-glucoside | 3.77 | 0.28 |  | 3.83 | 0.45 |
| 2 | cyanidin 3-*O*-glucoside | 1.65 | 0.67 |  | 1.63 | 1.49 |
| 3 | petunidin 3-*O*-glucoside | 2.94 | 0.35 |  | 3.00 | 1.01 |
| 4 | peonidin 3-*O*-glucoside | 2.26 | 0.03 |  | 2.28 | 1.38 |
| 5 | malvidin 3-*O*-glucoside | 10.53 | 0.02 |  | 10.60 | 0.89 |
| 6 | myricetin 3-*O*-galactoside | 1.32 | 0.44 |  | 1.32 | 2.22 |
| 7 | myricetin-3-*O*-glucoside | 5.86 | 0.33 |  | 6.06 | 1.87 |
| 8 | quercetin 3-*O*-arabinopyranosyl- | 2.86 | 0.54 |  | 2.87 | 0.99 |
|  | (1→2)-galactopyranoside |  |  |  |  |  |
| 9 | myricetin 3-*O*-glucuronide | 5.31 | 0.58 |  | 5.62 | 0.43 |
| 10 | rutin | 9.47 | 0.45 |  | 10.11 | 3.35 |
| 11 | quercetin 3-*O*-galactoside | 7.16 | 0.32 |  | 7.24 | 3.06 |
| 12 | quercetin 3-*O*-glucoside | 18.39 | 0.24 |  | 18.89 | 0.73 |
| 13 | kaempferol 3-*O*-robinobioside | 1.03 | 0.88 |  | 1.09 | 2.20 |
| 14 | quercetin-3-*O*-glucuronide | 61.68 | 0.07 |  | 65.09 | 0.09 |
| 15 | kaempferol 3-*O*-galactoside | 17.10 | 0.67 |  | 16.46 | 0.73 |
| 16 | isorhamnetin 3-*O*-rutinoside | 5.92 | 0.55 |  | 6.45 | 1.49 |
| 17 | kaempferol 3-*O*-glucoside | 39.17 | 0.05 |  | 37.79 | 2.22 |
| 18 | syringetin 3-*O*- glucoside | 1.32 | 0.44 |  | 1.44 | 1.63 |
| 19 | isorhamnetin 3-*O*-glucoside | 8.32 | 0.33 |  | 8.24 | 0.99 |
| 20 | kaempferol 3-*O*-glucuronide | 28.59 | 0.03 |  | 27.63 | 0.56 |
| 21 | kaempferol 7-*O*-glucoside | 1.95 | 0.78 |  | 1.92 | 3.45 |
| 22 | diosmetin 7-*O*-hexose | 1.64 | 0.55 |  | 1.61 | 1.16 |
| 23 | isorhamnetin 3-*O*-glucuronide | 1.15 | 0.39 |  | 1.19 | 1.42 |
| 24 | quercetin | 0.59 | 0.37 |  | 0.58 | 0.67 |
| 25 | diosmetin | 1.25 | 0.74 |  | 1.30 | 2.65 |

^1^ The numbers assigned to compounds correspond to those used in Table 2.

^2^ Content of compounds 1, 2, 10, 11, 12, 17, and 19 were quantified by comparison with external standards, while compounds 3, 4, 5 are given in mg/100 g FW equivalent of malvidin 3,5-diglucoside chloride. The other non-anthocyanin flavonoids were quantified as rutin.

^3^ RSD = (SD/mean) × 100
